# Failure or future? Exploring alternative antibacterials: a comparative analysis of antibiotics and naturally derived biopolymers

**DOI:** 10.3389/fmicb.2025.1526250

**Published:** 2025-02-03

**Authors:** Artemijs Sceglovs, Ingus Skadins, Marco Chitto, Juta Kroica, Kristine Salma-Ancane

**Affiliations:** ^1^Institute of Biomaterials and Bioengineering, Faculty of Natural Sciences and Technology, Riga Technical University, Riga, Latvia; ^2^Baltic Biomaterials Centre of Excellence, Headquarters at Riga Technical University, Riga, Latvia; ^3^Department of Biology and Microbiology, Riga Stradins University, Riga, Latvia; ^4^AO Research Institute Davos, Davos, Switzerland

**Keywords:** antibacterial naturally derived biopolymers, antibiotics, antibiotic resistance, bacterial infections, mechanism of action

## Abstract

The global crisis of antimicrobial resistance (AMR) is escalating due to the misuse and overuse of antibiotics, the slow development of new therapies, and the rise of multidrug-resistant (MDR) infections. Traditional antibiotic treatments face limitations, including the development of resistance, disruption of the microbiota, adverse side effects, and environmental impact, emphasizing the urgent need for innovative alternative antibacterial strategies. This review critically examines naturally derived biopolymers with intrinsic (essential feature) antibacterial properties as a sustainable, next-generation alternative to traditional antibiotics. These biopolymers may address bacterial resistance uniquely by disrupting bacterial membranes rather than cellular functions, potentially reducing microbiota interference. Through a comparative analysis of the mechanisms and applications of antibiotics and antibacterial naturally derived biopolymers, this review highlights the potential of such biopolymers to address AMR while supporting human and environmental health.

## Graphical abstract

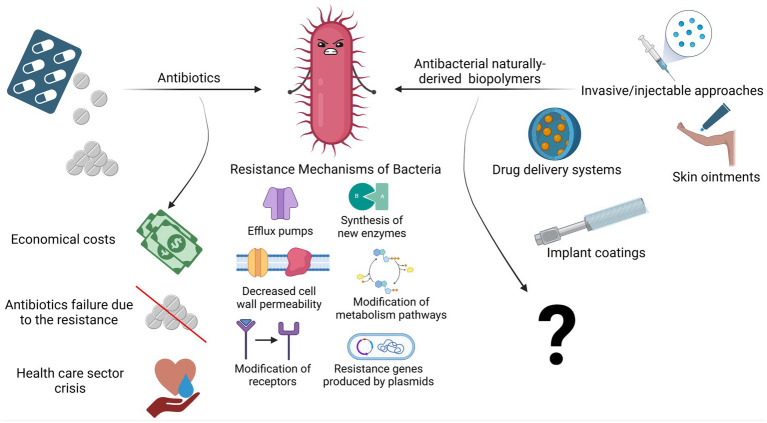

## 1 Introduction

The World Health Organization (WHO) estimates that antimicrobial resistance (AMR) could cause up to 10 million deaths annually by 2050, with a severe impact on global healthcare costs and economic stability. Bacterial infections are among the most life-threatening healthcare challenges, accounting for approximately 13.6% of global mortality and affecting 1 in 8 individuals worldwide (Appanna, 2018). The rise of multidrug-resistant (MDR) bacteria further highlights an urgent need for alternative, next-generation antibacterial treatments. While antibiotics have historically revolutionized healthcare, their widespread use has led to substantial challenges, including disrupting human microbiota and the rise of antibiotic-resistant bacterial strains.

Beyond their pathogenic roles, bacteria are integral to human health, especially in the gut, contributing to immune modulation and digestion (Bull and Plummer, 2014; Yin et al., 2019; Arciola et al., 2018). Consequently, the modern healthcare system cannot fully eliminate infection risk, particularly in post-surgical settings (Oliva et al., 2021; Hedrick et al., 2006; van Seventer and Hochberg, 2017) and procedures involving biomaterials or medical devices (Arciola et al., 2018; Oliva et al., 2021; Hedrick et al., 2006). Infections occur when infectious agents enter human body tissues, multiply, and trigger host immune responses (van Seventer and Hochberg, 2017). Although infections cannot be entirely prevented due to inevitable interactions with environmental microbes, targeted measures can mitigate bacterial invasion and inhibit replication at potential infection sites.

The human microbiome, especially the gut microbiota, comprises numerous symbiotic bacterial species (e.g., *Lactobacillus*, *Bacillus*, *Clostridium*, *Enterococcus*, and *Ruminococcus*) that collectively represent approximately 90% of the gut flora (Rinninella et al., 2019; Martín et al., 2013; Eloe-Fadrosh and Rasko, 2013). These beneficial microorganisms are crucial in digestion, nutrient absorption, and immune defense. Importantly, they maintain a delicate balance, contributing to immune regulation and protecting against pathogens without causing harm to the host (Rinninella et al., 2019; Bogitsh et al., 2019; Hemarajata and Versalovic, 2013; Isolauri et al., 2001; Wieërs et al., 2020; Hills et al., 2019; Ding et al., 2019). However, the broad-spectrum use of antibiotics has disrupted this balance, weakening the microbiota’s natural protective functions and impairing the immune response, leading to gut dysbiosis—an environment conducive to antibiotic-resistant strains (Patangia et al., 2022).

While antibiotics effectively eliminate harmful pathogens, their indiscriminate targeting also affects beneficial bacteria, reducing microbial diversity and increasing the likelihood of antibiotic resistance (Lathakumari et al., 2024). Inappropriate antibiotic use across sectors, including clinical and animal health, has further escalated the global antibiotic resistance crisis, contributing to the emergence of MDR pathogens that resist multiple antibiotic classes (Yang et al., 2024).

The decreasing efficacy of antibiotics and the limited availability of alternative treatments underscore the urgent need for new classes of antibacterial therapeutics. Ideal alternatives would incorporate mechanisms of action that lower the risk of resistance. In this context, naturally derived biopolymers (NDBs) have attracted significant attention due to their unique antibacterial properties. Although long used in biomedical applications, interest in biopolymers as antibacterial agents has surged, with publications on their use rising by approximately 400% since 2015 (Web of Science, n.d.).

Approximately two decades ago, antibacterial biopolymers, e.g., those with intrinsic antibacterial activity, were first proposed as alternatives to antibiotics for treating bacterial infections (Muñoz-Bonilla and Fernández-García, 2012). Today, biopolymer-based strategies show potential for localized, non-antibiotic antibacterial applications that support the immune system and minimize impact on the natural microbiota. Such approaches could represent a sustainable innovation within modern healthcare.

Notably, NDBs disrupt bacterial membranes instead of targeting specific metabolic pathways, a mechanism less prone to resistance development (Bustamante-Torres et al., 2022; Kamaruzzaman et al., 2019). Numerous studies have documented the use of NDBs in biomedical devices, including drug delivery systems, contact lenses, and injectable cement, where they exhibit potent antibacterial activity and biocompatibility (Pahlevanzadeh et al., 2022; Sam et al., 2023; Coma, 2013). This review provides a comprehensive examination of the potential of antibacterial NDBs, analyzing recent literature to compare their effectiveness and applications with those of conventional antibiotics. By exploring the mechanisms, advantages, and limitations of NDBs, this review assesses whether these biopolymers could serve as reliable, antibiotic-free therapeutics capable of complementing or partially replacing traditional antibiotics in treating bacterial infections—or whether their promise remains largely theoretical.

## 2 Antibiotics and antibiotic resistance

**Figure 1:**
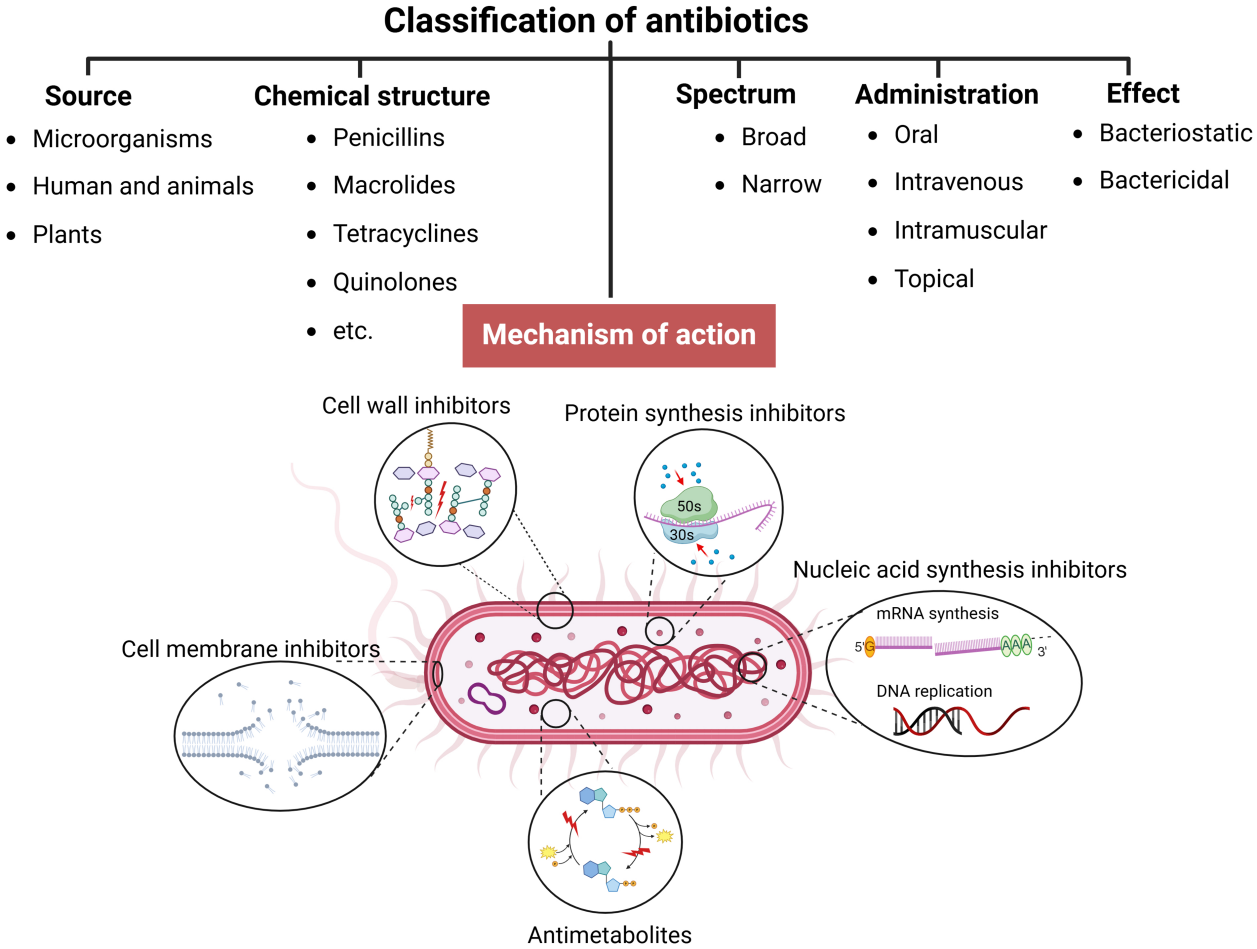
Classification of antibiotics (created by Biorender).

In the pre-antibiotic era, more than half of deaths were attributable to infections (Aminov, 2010). Since the 20th century, antibiotics have revolutionized antibacterial therapeutics in the history of medicine, drastically changing modern medicine and extending the average human lifespan (Johnston and Badran, 2022; Cook and Wright, 2022; Sikdar et al., 2021). Several groups and generations of antibiotics have been discovered and developed with specific target mechanisms of action on bacterial cells (Kapoor et al., 2017; Coates et al., 2011) (Table 1). Conventionally, antibiotics are classified as cell wall inhibitors, protein synthesis inhibitors, nucleic acid synthesis inhibitors, antimetabolites, and cytoplasmic membrane inhibitors (Pancu et al., 2021; Ullah and Ali, 2017) (Figure 1).

**Table 1:** Classification of antibiotics based on the mechanism of action and chemical structure with characterization: action mechanisms, reported side effects, and bacterial resistance mechanisms.

Classification group	Chemical structure	Group example/−s	Action mechanism	Side effects	Bacterial resistance mechanism	Reference
Cell wall inhibitors	β-Lactams	Penicillins Cephalosporins Carbapenems Monobactams	Disrupt peptidoglycan synthesis in the bacterial cell wall by binding to a transpeptidase enzyme	Allergic reaction	Production of the β-lactamase enzyme. Consequently, β-lactam antibiotic therapies also include additional drugs called β-lactamase inhibitors (clavulanate, sulbactam, and tazobactam) to block this enzyme action	Majiduddin et al. (2002), Waley (1992), Castle (2007), Arer and Kar (2023), Tehrani and Martin (2018), Bush and Bradford (2016), Romano et al. (2003), Iuliano et al. (2022), Solensky (2003)
	Glycopeptides	Vancomycin				
	Bacitracin	Bacitracin				
	Fosfomycin	Fosfomycin				
Protein synthesis inhibitors (50S ribosomes)	Macrolides	Erythromycin Azithromycin Clarithromycin	Bind to 50S/30S ribosomal subunits, inhibiting their function and preventing the synthesis of new proteins. The bacteriostatic or bactericidal effects of protein synthesis inhibitors depend on the dosage.	Dysbiosis, nephrotic syndrome, aplastic anemia, and others	Transcription modification, efflux pumps, and gene mutation	Dunn and Zambraski (1980), Antibiotics Review (2010), Protein Synthesis Inhibitors-Definition (2023)
	Chloramphenicol	Chloramphenicol Levomycetin				
	Linezolid	Linezolid				
	Clindamycin	Clindamycin				
Protein synthesis inhibitors (30S ribosomes)	Aminoglycosides	Amikacin Tobramycin Neomycin Gentamicin Streptomycin				
	Tetracyclines	Tetracycline Doxycycline Minocycline				
Nucleic acid synthesis inhibitors	Quinolones	Ciprofloxacin Norfloxacin Moxifloxacin Levofloxacin	Stabilizing the enzyme–DNA complex and thus interrupting the relegation step	Aortic dissection, tendinitis, and hepatotoxicity	Modification of two enzymes: DNA gyrase and topoisomerase IV	Kapoor et al. (2017), Bhattacharjee (2016), Collin et al. (2011), Ramappa and Aithal (2013)
	Rifamycin	Rifampicin	Bind to RNA polymerases, thus blocking RNA synthesis		RNA polymerase mutation	
Antimetabolites	Sulfanilamides	Sulfamethoxazole	Inhibits folic acid synthesis in bacteria, a crucial element for DNA synthesis	Weight loss, weakness, and mouth inflammation	Efflux pumps and enzymatic inactivation	Chortkoff and Stenehjem (2019), McGee et al. (2018), Hanlon et al. (2019)
	Dihydrofolate reductase inhibitors	Trimethoprim				
Cell membrane inhibitors	Polymyxins	Colistin	Target phospholipids in the cell membrane, thus altering membranes’ physical properties	No reported data, as minimal clinical applications	Increase in drug efflux, mutation, and alteration of the porin pathway	Cell Membrane Inhibitors (2023)
	Daptomycin	Daptomycin				

Other classification principles of antibiotics rely on their origin, spectrum of action, administration strategies, chemical structure, and mechanism of action (Figure 1). Antibiotics can be administered through various routes, including oral, intravenous, intramuscular, and topical applications (Buonavoglia et al., 2021; Enenkel and Stille, 1988). The topical application is particularly relevant for localized infections, such as skin wounds or mucosal infections, where direct delivery to the affected area can enhance efficacy and minimize systemic side effects. The effectiveness of these administration strategies depends on various factors, including bioavailability, drug formulation, gastrointestinal conditions, and systemic distribution, which, if not optimized, could compromise therapeutic outcomes and limit the antibiotic’s efficacy against targeted infections (McCarthy and Avent, 2020; Vinarov et al., 2021). Moreover, antibiotics exhibit specific behavioral characteristics, including whether they are bactericidal or bacteriostatic, as well as their spectrum, which can be broad or narrow. Antibiotics with a wide spectrum and bactericidal action may impact the microbiota within organisms’ niches (Dubourg et al., 2014; Blaser, 2011; Yang et al., 2021), resulting in dysbacteriosis conditions post-therapy and an increasing risk of secondary disease. In addition, the systemic use of antibiotics has been documented to affect various organ systems, leading to heightened organism toxicity (Berry et al., 1995; Grill and Maganti, 2011) (Table 1).

**Figure 2:**
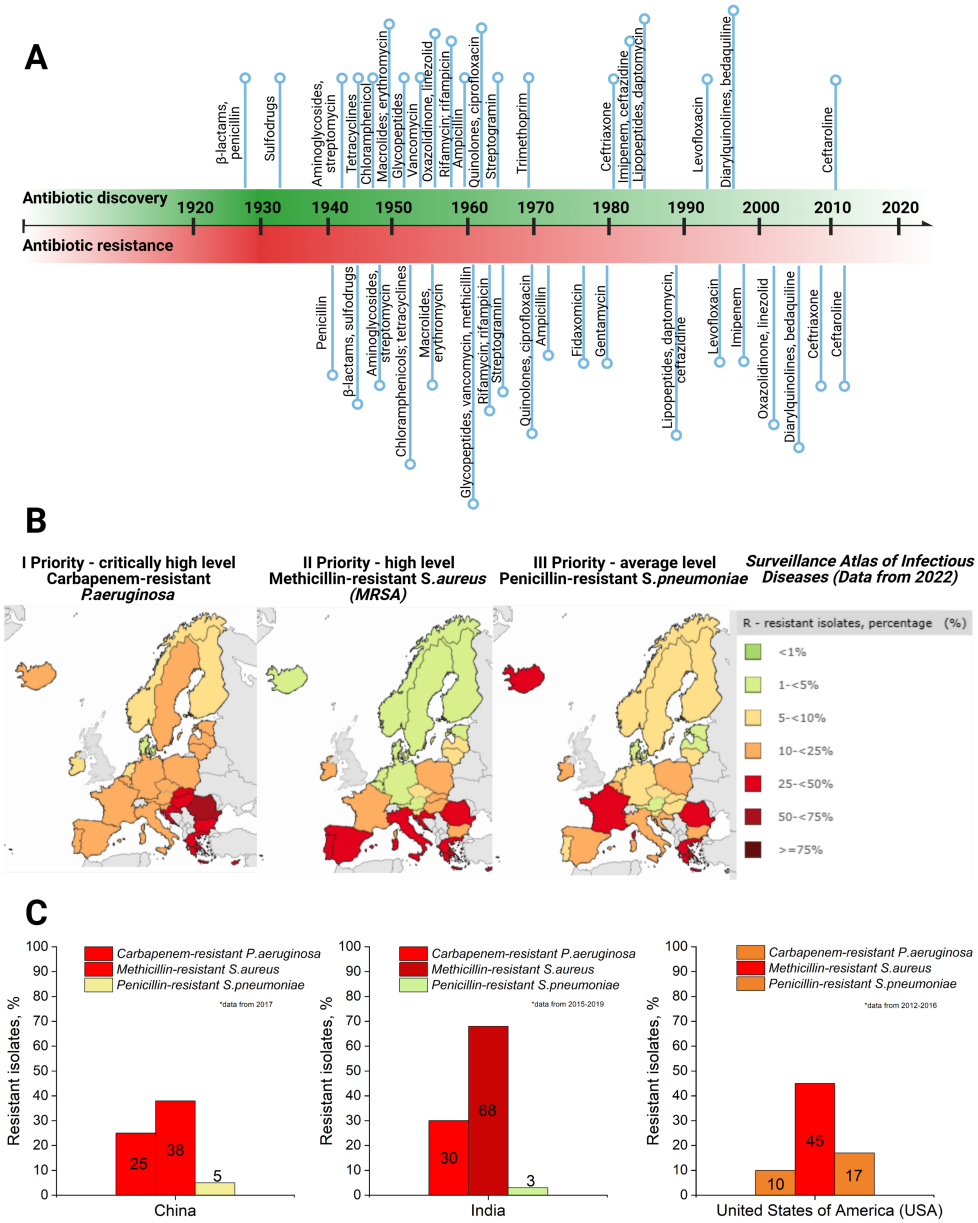
**(A)** Antibiotic discovery (title of antibiotics and relative point within timeline decade) and resistance reports timeline (resistance report against specific antibiotic with a title and relative point within timeline decade) (Dahal and Chaudhary, 2018). **(B)** Case map on three priority levels (I priority stands for critically high level; II priority – high level, and III – average level of resistance cases) resistant strains (carbapenem-resistant *P. aeruginosa*; methicillin-resistant *S. aureus*, and penicillin-resistant *S. pneumoniae*) in Europe, 2022, as well as a supporting table on resistant isolates’ case percentage and colorful indicator for priority, with green indicating low level and dark red indicating critically high level (Surveillance Atlas of Infectious Diseases, 2022). **(C)** Comparative graphs of the same three priority resistant strains with color indication in the top three most populated world countries: China, India, and the United States of America (USA) (ResistanceMap, n.d.) (created by Biorender).

Currently, bacteriophage therapy is the only alternative as effective as antibiotics. Phage therapy relies on using naturally occurring bacteriophages (viruses) to infect and lyse bacteria at the site of infection (Lin et al., 2017). However, phage therapy must still be licensed in the majority of countries or used under exceptional situations (Yang et al., 2023). Thus, antibiotics remain the primary treatment option in clinics to combat bacterial infections. However, the development of antibiotics has begun an endless race against pathogenic microorganisms. As a side problem, the overuse and misuse of these lifesaving drugs have developed the top global public health crisis named antibiotic resistance occurring worldwide (Akram et al., 2023). Antibiotic resistance arose from the evolutionary development of primary (antibiotic target site is not presented in bacteria strain) and secondary (genome-related and plasmid-related) resistance mechanisms in bacteria (Urban-Chmiel et al., 2022; Nilsson, 2019; Zhang and Cheng, 2022) (Table 1). The dramatic report by the WHO has shown that by 2050, drug resistance could catch up to cancer and sufficiently damage the economy if actions are not taken (World Health Organization, 2019). The uncontrolled use of antibiotics in agriculture and inappropriate therapeutic practices has provided an evolutionary advantage to bacteria, such as methicillin-resistant *Staphylococcus aureus* (MRSA), vancomycin-resistant *Enterococcus* (VRE), and others that have become resistant to one or multiple types of antibiotics, contributing to the dramatic situation in healthcare (Figure 2).

The global threat of antimicrobial resistance (AMR) necessitates collaborative action to develop and implement effective strategies (Uchil et al., 2014). Several preventative measures have been established and continue to evolve, addressing AMR at international, national, community, hospital, and individual levels (Uchil et al., 2014). At the international level, efforts focus on enhancing collaboration among governments, non-governmental organizations, professional groups, and international agencies. Key initiatives include global networks for antimicrobial use and resistance surveillance, strategies to combat counterfeit antimicrobials, and programs to foster innovation in new drugs and vaccines. Strengthening global AMR control programs remains a priority. Nationally, dedicated committees and AMR policies have been introduced to monitor and manage AMR. These policies integrate geographical, social, and economic factors to provide tailored solutions. Educational initiatives, including training programs and certification courses, aim to equip healthcare professionals and the private sector with knowledge for the rational use of antibiotics. Regulatory controls to limit over-the-counter antibiotic sales further address misuse, a key driver of resistance. For example, a review revealed that non-prescription antibiotic use varies widely, from 3% in Northern Europe to 100% in some African regions (Uchil et al., 2014; Morgan et al., 2011). In addition, efforts are directed at improving standards in healthcare systems, microbiology laboratories, and pharmaceutical companies. Protecting existing antibiotic therapies remains critical, with ongoing research focused on developing new drugs to replace outdated ones and prolonging the effectiveness of current treatments. It has already been proven that synergy and drug combinations are a winning strategy in fighting multidrug-resistant bacteria and might help protect the existing drugs through antibiotic adjuvants. For instance, *β*-lactamase inhibitors have been used as adjuvants for penicillin group antibiotics as they block the resistance mechanism of bacteria against these antibiotics (see Table 1). Other examples include efflux pump inhibitors and outer membrane permeabilizers (Annunziato, 2019).

## 3 Naturally derived biopolymers with intrinsic antibacterial properties

Naturally derived biopolymers (NDBs) are large macromolecules from living organisms such as plants and microorganisms. These polymers are formed through enzyme-catalyzed chain-growth polymerization processes of activated monomers (Sun et al., 2022). The molecular size of NDBs varies significantly based on their type and source, ranging from a few kilodaltons (kDa), as seen in polysaccharides such as chitosan (~10–50 kDa), to several megadaltons (MDa), such as cellulose and other structural polysaccharides (>1 MDa) (Moradali and Rehm, 2020; Yadav et al., 2015). This broad size range supports their diverse physicochemical properties and wide-ranging applications (Troy et al., 2021; Reddy et al., 2021).

The reason is that the source of these compounds is derived from living organisms through enzymatic polymerization, forming high molecular weight macromolecules. As a result, covalently bonded repetitive monomeric units form biodegradable compounds such as polysaccharides, polyamino acids, hydroxy fatty acids, polypeptides, and glycolipids (Moradali and Rehm, 2020; Yadav et al., 2015; Troy et al., 2021) (see Table 2). These compounds are classified as NDBs and have unique physical, chemical, and mechanical properties, which are exploited in biomedical applications. NDBs are commonly used in the development of drug delivery systems (Baranwal et al., 2022; Murali and Jayakumar, 2023; Atanase, 2021). In addition, NDBs such as collagen, gelatin, dextran, agarose/alginate, hyaluronic acid, cellulose, and fibrin are also being explored in various other biomedical applications, including open incision/wound suturing, fixing, adhesion, covering, occlusion, isolation, contact inhibition, cell proliferation, tissue guiding, and controlled drug administration (Baranwal et al., 2022). NDBs are of broad interest because of their potential to be used for developing environmentally friendly medical devices that perform high biocompatibility and serve as highly accurate biosensors, drug delivery systems, etc. (Manoukian et al., 2019). In addition to biocompatibility, biodegradation, bioadhesiveness, and biofunctionality of the NDBs, several drawbacks must be addressed, such as low stability, low melting point, high surface tension, structural complexity, and well-known immunological response from organisms (Ige et al., 2012; Jenkins et al., 1996; Reddy et al., 2021). Various NDBs such as chitosan, pectin, *κ*-carrageenan, alginate, *ε*-polylysine, and others have also been identified for their antibacterial activity (Muñoz-Bonilla et al., 2019; Li et al., 2021; Habeeb and Abdulkadhim, 2024; Hamidi et al., 2023) (see Table 2). Numerous studies have demonstrated the comparative effectiveness and potential advantages of NDBs over conventional antibiotics. For instance, Tin et al. (2009) reported that chitosan molecules with different molecular weights consistently exhibited a minimum inhibitory concentration (MIC) of 32 μg/mL against various strains of *P. aeruginosa*. In contrast, the MIC range for sulfamethoxazole was significantly broader, ranging from 64 to 2048 μg/mL (Tin et al., 2009). Similarly, Si et al. (2021) found that a chitosan derivative effectively inhibited Gram-negative and Gram-positive bacteria, with MIC values ranging from 8 to 32 μg/mL. Specifically, against *A. baumannii*, the chitosan derivative achieved an MIC of 32 μg/mL, compared to higher MIC values of 128 μg/mL for amikacin and tobramycin and 64 μg/mL for tazobactam. However, certain antibiotics outperformed NDBs in specific cases; for example, novobiocin demonstrated an MIC of 8 μg/mL, and carbenicillin and tobramycin were more effective against MRSA (Si et al., 2021). Another noteworthy example is ε-polylysine, which exhibited MIC values of 500, 800, 800, and 1,000 μg/mL against *P. aeruginosa*, *K. pneumoniae*, MSSA, and MRSA, respectively. Traditional antibiotics such as ampicillin, gentamicin, and tetracycline showed MIC values ranging from 35 to 250 μg/mL against the same bacterial strains (Sundaran et al., 2022). Importantly, combining NDBs with antibiotics has synergistic effects, significantly enhancing antibacterial activity and reducing the required antibiotic dosage (Si et al., 2021; Taheri-Araghi, 2024; Fayed et al., 2023; Baltimore et al., 1987; Khan et al., 2012; Kaur et al., 2022).

**Table 2:** Examples of naturally derived biopolymers (NDBs) and their antibacterial performance.

Class of biomolecule	Examples	Source	Intrinsic antibacterial activity (modification examples for improvement)	Antibacterial mechanism (bacteriostatic/bactericidal effect)	Reference
Polysaccharides	Chitin	Invertebrate animals (crustaceans)	+ (modified into chitosan form)	Makes the bacteria flocculate and thus kill it, presumably through lack of nutrients and oxygen (i.e., mass transfer limitation)	Kucharska et al. (2019), Benhabiles et al. (2012)
	Chitosan	Invertebrate animals (crustaceans) and certain fungi	+ (modified with quaternary ammonium)	Membrane disruption by electrostatic interaction	Muñoz-Bonilla et al. (2019), Razak and Mohamed (2021)
	Cellulose	Plants	− (modified with essential oils, metal nanoparticles, quaternary amino groups, etc.)	–	Muñoz-Bonilla et al. (2019), Nemeş et al. (2022)
	Starch	Plants	− (modified with metal oxides, antimicrobial peptides, essential oils, etc.)	–	Hou et al. (2023)
	Alginate	Macroalgae	+ (modified with essential oils, peptides, and metal nanoparticles)	Membrane disruption by electrostatic interaction	Wathoni et al. (2024), Asadpoor et al. (2021), Hegde et al. (2022)
	Pectin	Plants	+ (modified with peptides, metal nanoparticles, antibiotics, and metal ions)	Still unclear, molecules cause double oxidative stress	Muñoz-Bonilla et al. (2019), Tripathi and Mishra (2021), Daoud et al. (2013), Hassan et al. (2021)
	κ-Carrageenan	Macroalgae	+ (modified with metal oxides, metal nanoparticles, essential oils, and clay)	Damages the bacterial cell wall and cytoplasmic membrane and suppresses the growth of both Gram-positive and Gram-negative bacteria	Muñoz-Bonilla et al. (2019), Zhu et al. (2017), El-Fawal (2014)
	Chondroitin sulfate	Humans, other mammals, invertebrates, and some bacteria	+ (modified with chitosan or zinc ions)	Membrane disruption by electrostatic interaction	Unver et al. (2023), Gómez et al. (2018), Wu et al. (2022)
	β-glucans (laminaran, scleroglucan etc.)	Fungi, yeasts, and algae	+ (modified with zinc oxide, enzyme proteins, or carboxymethylated)	Penetrates bacterial cells, interfering with their metabolism and inducing cellular lysis	Chamidah and Hardoko (2017), Schwartz and Vetvicka (2021), Pino et al. (2023), Syaban et al. (2022), Song et al. (2020)
	o-Pullulan	Fungi	+ (modified with silver zinc oxide nanoparticles)	Membrane disruption by electrostatic interaction	Rai et al. (2021), Roy et al. (2023)
	Fucoidan	Brown algae	+ (modified with other molecules, e.g., chitosan and collagen)	Binds with the bacterial DNA, cytoplasmic membrane, and compounds present in the cell wall of bacteria and leads to the leakage of protein and an increase of the cytoplasmic membrane permeability, which results in the antibacterial effect of fucoidans	Habibi et al. (2024), Chmit et al. (2014), Egle et al. (2024)
Exo-polysaccharides	Hyaluronan	Bacteria	+ (modified with peptides, amino acids, and other polysaccharides, e.g., chitosan)	Neutralizes positive charge of the bacterial cell wall and so dramatically compromises bacteria adhesion ability	Zamboni et al. (2023), Hernandez-Montelongo et al. (2021)
	Xanthan	Bacteria	−(biodegraded into xanthan-oligosaccharide, modified with metal oxides)	–	Wang et al. (2020), Guo et al. (2022)
	Curdlan	Bacteria	− (modified with polyphenols and quaternary ammonium)	–	Suflet et al. (2024), Ding et al. (2024)
Proteins	Collagen	Animals and marine organisms	− (modified into oxidized form, carboxymethylated, with chitosan, alginate, antibiotics, herbal extracts, metal oxides, and peptides)	–	Valenzuela-Rojo et al. (2020), Ersanli et al. (2023)
	Silk (silk fibroin)	Silkworms	− (modified with antibiotics, inorganic nanoparticles, plant extracts, nitric oxide, and peptides)	–	Kaur et al. (2014), González-Restrepo et al. (2024), Ghalei and Handa (2022)
	Keratin	Animals	− (modified with metal nanoparticles, amides, and collagen)	–	Shanmugasundaram and Ramkumar (2018), Sun et al. (2023)
	Lactoferrin	Milk and colostrum	+ (modified with polyphenols, chitosan, and alginate)	Iron sequestering and further interaction with the bacterial surface lead to damaging the bacterial membrane, altering the outer membrane permeability	Jenssen and Hancock (2009), Wang et al. (2024)
	Lysozyme	Majority of vertebrates, including mammals	+ (modified with silica)	Cell wall disruption by hydrolyzing of 1,4-beta-linkages between *N*-acetylmuramic acid and *N*-acetylglucosamine	Khorshidian et al. (2022), van den Heuvel et al. (2018)
	Fibrin	Blood plasma of animals	− (used in combination with growth factors and other biological molecules in the form of platelet-or leukocyte-rich fibrin)	–	Moraschini et al. (2024)
Peptides (small amino acid-based biopolymers)	Magainin 2	Tailless amphibians	+ (modified with other cationic peptides)	Membrane disruption by electrostatic interaction	Kim et al. (2018), Syryamina et al. (2024)
	Defensins	Plants, insects, and mammals	+ (modified with chitosan and polylactic co-glycolic acid)	Membrane disruption by forming channels in lipid bilayer	Dong et al. (2020)
	LL-37 (Cathelin-associated antimicrobial peptide)	Neutrophils and macrophages in mammals	+ (modified with polylactic co-glycolic acid)	Membrane disruption by electrostatic interaction	Ren et al. (2024), Ridyard and Overhage (2021)
	Nisin	Bacteria	+ (modified with polysaccharides, proteins, calcium phosphates, and metal oxides)	Pore formation in the membrane and inhibition of cell wall biosynthesis by binding to lipid II	Shin et al. (2016), Li et al. (2018), Yan et al. (2024)
	Cecropin A	Insects	+ (no data)	Aggregate and assume a transbilayer orientation in membranes	Silvestro et al. (2000)
	ε-Polylysine	Bacteria	+ (modified with natural and synthetic polymers)	Membrane disruption by electrostatic interaction	Ranjbar et al. (2023), Sceglovs et al. (2023)
Other biopolymers	Suberin	Plants	+ (modified with essential oils)	Disruption of the bacterial membrane, prevention of biofilm formation, and inhibition of DNA and protein synthesis	Liakos et al. (2019), Dönmez and Önem (2024)
	Tannin	Plants	+ (used as a natural cross-linking agent for natural and synthetic polymers)	Iron chelation, inhibition of cell wall synthesis, and disruption of cell membrane	Farha et al. (2020), Baldwin and Booth (2022)

Considering the advantageous functionalities such as biodegradability, low immunogenicity, and non-toxicity of naturally derived biopolymer-based drug delivery systems, the antibacterial feature opens new horizons for developing local targeted antibacterial therapeutics based on antibacterial biopolymers. Countless reviews and studies have demonstrated the ability of NDBs to inhibit a broad spectrum of Gram-positive and Gram-negative bacteria, including bacterial strains currently being classified as “under urgent attention” due to their resistance to various antibiotics (Bustamante-Torres et al., 2022; Poznanski et al., 2023; Rofeal et al., 2022), as well as fungi (Poznanski et al., 2023; Ntow-Boahene et al., 2021) and viruses (Akbari et al., 2022; Bianculli et al., 2020). Several studies have highlighted that various naturally derived biopolymers (NDBs) exhibit notable antibiofilm activity (see Table 2). These antibiofilm mechanisms primarily involve disrupting biofilm exopolysaccharides (EPS), a critical component for biofilm stability. Such disruptions can lead to the detachment of bacterial cells or inhibit bacterial adhesion during the early stages of biofilm formation (Mishra et al., 2020; Melander et al., 2020). In addition, certain NDBs, such as lactoferrin-derived peptides, neutrophil peptides, and antimicrobial peptides (e.g., protegrin-1), have demonstrated antibacterial activity against intracellular pathogens, including *Mycobacterium tuberculosis*. These antibacterial effects are attributed to the disruption of the mycobacterial cell wall and enhanced membrane permeabilization (Khara et al., 2020; Jacobo-Delgado et al., 2023; Intorasoot et al., 2022). Despite these promising findings, significant challenges remain in translating NDBs into clinical applications. Key limitations include variability in their physicochemical and mechanical properties, which can impact reproducibility and reliability in therapeutic settings (Moradali and Rehm, 2020; Yadav et al., 2015). The limited physicochemical stability and difficulty tuning their biodegradation profiles further complicate their development as viable therapeutic solutions (Muñoz-Bonilla and Fernández-García, 2012; Pahlevanzadeh et al., 2022). In addition, the transition from laboratory-scale research to clinical application faces substantial barriers, including extensive preclinical testing to establish safety and efficacy, the complexities of large-scale manufacturing to ensure consistent quality, and the rigorous regulatory approval processes that demand considerable time and resources (Oliva et al., 2021; Murali and Jayakumar, 2023). Addressing these challenges will require interdisciplinary approaches and sustained efforts to optimize the properties of NDBs and streamline their development pipeline for clinical use (Arciola et al., 2018; Kong et al., 2023).

In further sections, the antibacterial potential of NDBs will be discussed to understand biopolymer interaction with bacterial cells, inhibition/bactericidal mechanism, and application specifics, to compare all these aspects with currently used conventional antibiotics, and to address the question posed in the title of this review.

## 4 Mode of delivery of NDBs for antibacterial treatment

**Figure 3:**
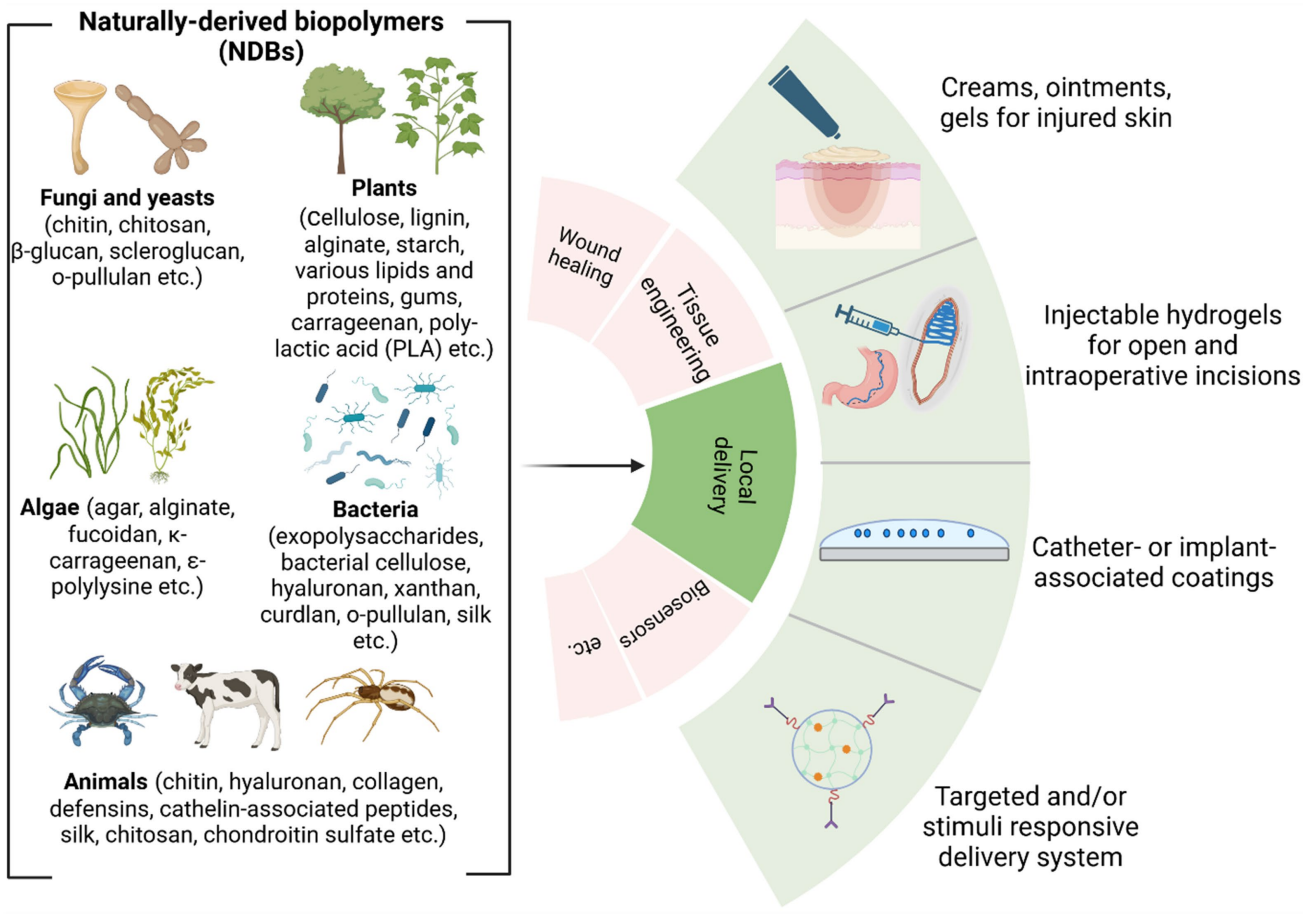
Overview of naturally derived biopolymers for local delivery applications (Biswas et al., 2022; Dimri et al., 2023; Ibrahim et al., 2019) (created by Biorender).

Based on R&D reports, the most common local modes of delivery of antibacterial naturally derived biopolymers (aNDBs) to treat desired sites for various biomedical applications are summarized in Figure 3. Local application options and antibacterial activity are the main advantages reported in numerous studies for such biopolymers (Wu et al., 2022; Jarosz et al., 2023; Kong et al., 2023). Local delivery is preferable as it achieves the target site at the same concentration as it was prepared directly without passing through all the body barriers via the bloodstream and without losing biopolymer molecules. Such local delivery types include creams, ointments, and gels that are applied on the skin, burn or opened wounds, and surgery sites to prevent and treat infection (Zhao et al., 2023); intraoperative coatings and fillers (Ilić-Stojanović et al., 2023) are used for deeper surgical sites or dental sockets post-tooth extraction to avoid or combat already infected site; implant and catheters coating that allows preventing implant-and catheter-associated infections (Veiga and Schneider, 2013); and controlled delivery systems that achieve and bind to targeted site via different stimuli or due to specific conditions (temperature and pH), followed by antibacterial activity while providing controlled cell/ion/growth factor release from the matrix (Jacob et al., 2018) (Figure 3). This local delivery feature gives an advantage compared to conventional administration of antibiotics orally (pills and suspensions) or intravenously (in particular cases). While oral administration is convenient and suitable for at-home antibiotic therapy, it is associated with a decline in the concentration of the active compound upon reaching the infection site (Homayun et al., 2019; Kim and De Jesus, 2023). In addition, this strategy negatively impacts natural body microbiota, directly affecting the patient’s immune system response to continuous or new bacteria invasion (Konstantinidis et al., 2020). Nevertheless, it is worth acknowledging the efficacy of oral drug administration in systemic or severe infections at multiple sites (Cunha, 2006). However, various studies have reported that combined device development where aNDBs served as a drug delivery system with encapsulated antibiotics might have a promising synergistic effect to achieve the exact infection site (Liu et al., 2022; Khan et al., 2021; Meng et al., 2014; Hwang et al., 2023; Schrade et al., 2022).

## 5 Mechanism of antibacterial action

**Figure 4:**
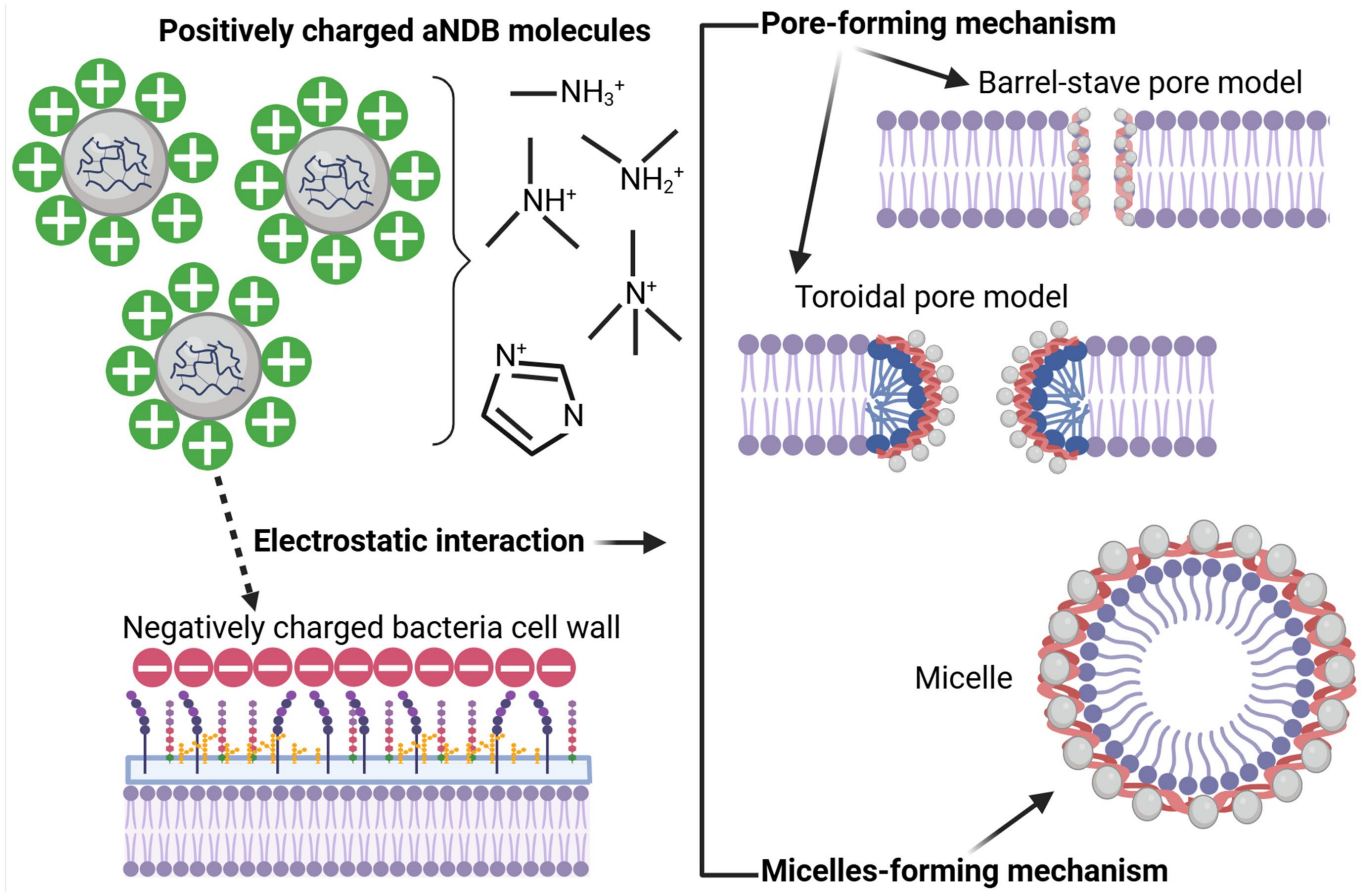
Biopolymer (aNDBs)—bacterial cell interaction (left) and bactericidal mechanisms (right) (created by Biorender).

Regarding aNDBs, it is crucial to understand that these biopolymer molecules, unlike the previously described antibiotics, do not target specific synthesis pathways or molecules. First, it is worth mentioning that bacterial cell wall outer structures serve as adhesion and pathogenicity factors; for example, lipopolysaccharides and phospholipids of Gram-negative bacteria and teichoic and lipoteichoic acids of Gram-positive bacteria are negatively charged. Second, aNDBs consist of positively charged molecules (chondroitin sulfate, o-pullulan, alginate, *ε*-polylysine, chitosan, magainin-2, etc.), containing cationic groups such as quaternary ammonium, quaternary phosphonium, guanidinium, or tertiary sulfonium (Santos et al., 2016), which have a positive charge. As a result, the interaction between biopolymers and bacteria begins with mutual attachment caused by electrostatic forces (Haktaniyan and Bradley, 2022). As a result, if aNDB molecules and bacteria cells are close enough, oppositely charged molecules attract each other, leading to physical binding (Figure 4). Another essential fact is that not all cationic molecules are lethal to bacteria. The electrostatic interaction represents just the first step toward the bactericidal effect of aNDBs. Second, a specific concentration of the cationicity of aNDBs must be achieved to reach a multivalence effect (Smola-Dmochowska et al., 2023) that results in the simultaneous binding of aNDB molecules to the bacterial cell structures and moving to the next step.

In the next step, aNDB mechanisms of action on bacterial cell walls are divided into pore-forming and micelle-forming mechanisms (Qiu et al., 2020; Zhou et al., 2023). The pore-forming mechanism can be further categorized into two models: barrel-stave pore and toroidal pore (Kumar et al., 2018; Pastore et al., 2020; Mihajlovic and Lazaridis, 2010). Within the barrel-stave model, the aNDB molecules are initially oriented parallel to the membrane but eventually inserted perpendicularly in the lipid bilayer (Hegde et al., 2022) (Figure 4). This promotes lateral peptide–peptide interactions such as membrane protein ion channels. Hydrophobic regions interact with membrane lipids, and hydrophilic residues form the lumen of the channels (Brogden, 2005). On the other hand, in the toroidal pore model, peptides are also inserted perpendicularly in the lipid bilayer, but specific peptide–peptide interactions are not present (Wimley, 2010). Instead, the peptides induce a local curvature of the lipid bilayer, with the pores partly formed by peptides and partly by the phospholipid head group. The dynamic and transient lipid–peptide supramolecule is the “toroidal pore.” The key distinguishing feature of this model, compared to the barrel-stave pore, is the net arrangement of the bilayer. In the barrel-stave pore, the hydrophobic and hydrophilic arrangement of the lipids is maintained, whereas, in toroidal pores, the hydrophobic and hydrophilic arrangement of the bilayer is disrupted. This provides alternate surfaces for interacting with the lipid tail and head group. As the pores are transient upon disintegration, some peptides translocate to the inner cytoplasmic leaflet, entering the cytoplasm and potentially targeting intracellular components (Kumar et al., 2018). Other features of the toroidal pores include ion selectivity and discrete size (Yeaman and Yount, 2003). Due to pore formation, joint cell wall integrity and permeability are disrupted, resulting in bacterial cell lysis.

The micelle-forming mechanism is usually called the “Carpet-like” model (Wimley, 2010; Huan et al., 2020; Shai, 2002). In this case, the aNDBs adsorb parallel to the lipid bilayer and reach a threshold concentration to cover the surface of the membrane, thereby forming a “carpet” (Figure 4). This leads to unfavorable interactions on the membrane surface. Consequently, membrane integrity is lost, producing a detergent-like effect, which eventually disintegrates the membrane by forming micelles, followed by bacterial cell death (Qiu et al., 2020; Zhou et al., 2023; Kumar et al., 2018).

## 6 Resistance development possibility

Another crucial consideration lies in the potential for bacteria to develop resistance to antibiotics. As previously highlighted, different bacterial strains develop resistance to commonly used antibiotics. Resistance mechanisms are unique and depend on the antibiotic group and mechanism of action specifics. Still, overall mechanisms involve specific enzyme production, loss of targeted molecules, efflux pumps, mutation of the target site, increased cell permeability, etc. (Reygaert, 2018; Munita and Arias, 2016; Peterson and Kaur, 2018; Abushaheen et al., 2020). It has been conventionally assumed that this propensity for resistance is exclusive to antibiotics, and theoretically, bacteria cannot develop resistance to aNDBs. On the one hand, electrostatic attraction between aNDBs and bacterial outer structures seems inevitable. In addition, the aNDB mechanism of action is not explicitly targeted. Even after entering the inner environment, aNDBs could enter many metabolic pathways. Based on that, it is more likely that bacteria encounter challenges in impeding electrostatic interaction and developing resistance, given the biological expense associated with such a complex process.

Although thought to be improbable, alteration of bacterial membranes has been shown as a mechanism of resistance (Epand et al., 1858; Nawrocki et al., 2014). Such alterations include incorporating components with reduced anionic charge, which leads to the inability of peptides to aggregate on bacterial membranes and prevents them from entering the cell (Baltzer and Brown, 2011). For instance, studies have shown that *Staphylococcus aureus* modifies the anionic phospholipids in the cytoplasmic membrane with L-lysine, resulting in a reduction of the net negative charge of the bacterial membrane and leading to the repulsion and subsequent resistance to aNDBs (Peschel et al., 2001). Similarly, modification of Gram-negative bacteria’s lipopolysaccharides (LPS) is another bacterial mechanism contributing to resistance (Gunn, 2001; Guo et al., 1998). These modifications include incorporating fatty acids, thereby reducing the permeability of the outer membrane and increasing membrane structural stability (Peschel, 2002). Furthermore, bacteria can change the permeability of the cell wall, as is widely reported in the case of tetracyclines (Chmit et al., 2014); in addition, such non-specific structures as efflux pumps are also responsible for pumping out unfavorable molecules, and they are evidenced to work correctly against macrolides (Zhong and Shortridge, 2000).

## 7 Conclusion and future perspective

The highlighted findings in our review confirm that naturally occurring biopolymers with intrinsic antibacterial performance can be considered high-performance, sustainable, next-generation materials for biomedical field applications. Various studies have shown that hydrogels, biosensors, drug delivery systems, and implant coatings based on natural antibacterial biopolymers have promising physicochemical features. They possess excellent biocompatibility and are naturally derived, thus making them environmentally friendly. However, the main focus of this review was to elucidate the potential of naturally derived antibacterial biopolymers toward a specific aim—antibacterial therapy against bacterial infection in the human body—and second, to understand whether aNDBs are a future or a failure in replacing antibiotics. Multiple studies have reported these biopolymers *in vitro*; their antibacterial potential revealed action mechanisms. In addition, aNDBs have demonstrated substantial inhibitory effects against antibiotic-resistant bacteria strains. Based on the results, aNDBs could emerge as a novel weapon against bacterial infections to replace currently used antibiotics and antibiotic use approaches. Unique antibacterial action and the possibility of loading directly to the infection site (locally) open new horizons for this type of material.

However, learning from the past must be taken properly; many years ago, antibiotics were in the same situation. What is known for sure is that antibacterial biopolymers exhibit remarkable potential for combating bacteria and possess unique qualities. This material class is confined to research studies and is exclusively utilized for scientific purposes under controlled conditions. At the same time, antibiotics have already deserved the trust of medical doctors and have been proven effective antibacterial therapy in clinical care. Bacteria possess the biological mechanisms necessary for potential evolution, raising questions about the likelihood of encountering analogous issues. The problem associated with antibiotic resistance has emerged due to prolonged global exposure to antibiotics in the medical sector, inappropriate drug misuse or overuse, and the usage of antibiotics in agriculture. It is still being determined if antibacterial biopolymers will be opened to the world as much as antibiotics and undergo the same conditions. Will we face the same problem as now? Bacteria possess the biological mechanisms necessary for potential evolution, raising questions about the likelihood of encountering similar issues. In summary, antibacterial biopolymers are promising materials with many advantages, including their antibacterial potential. However, in light of various considerations and experiences, numerous questions must be answered, particularly considering the development of bacterial resistance. It is not merely a matter of substituting one for the other but a nuanced exploration of the complexities involved.
